# Bovine explant model of degeneration of the intervertebral disc

**DOI:** 10.1186/1471-2474-9-24

**Published:** 2008-02-25

**Authors:** Sally Roberts, Janis Menage, Sarit Sivan, Jill PG Urban

**Affiliations:** 1 Centre for Spinal Studies, Robert Jones and Agnes Hunt Orthopaedic Hospital, Oswestry, Shropshire SY10 7AG, UK & ISTM, Keele University, UK; 2 Department of Genetics, Anatomy and Physiology, Oxford University, Oxford OX1 3QX, UK

## Abstract

**Background:**

Many new treatments for degeneration of the intervertebral disc are being developed which can be delivered through a needle. These require testing in model systems before being used in human patients. Unfortunately, because of differences in anatomy, there are no ideal animal models of disc degeneration. Bovine explant model systems have many advantages but it is not possible to inject any significant volume into an intact disc. Therefore we have attempted to mimic disc degeneration in an explant bovine model via enzymatic digestion.

**Methods:**

Bovine coccygeal discs were incubated with different concentrations of the proteolytic enzymes, trypsin and papain, and maintained in culture for up to 3 weeks. A radio-opaque solution was injected to visualise cavities generated. Degenerative features were monitored histologically and biochemically (water and glycosaminoglycan content, via dimethylmethylene blue).

**Results and Conclusion:**

The central region of both papain and trypsin treated discs was macro- and microscopically fragmented, with severe loss of metachromasia. The integrity of the surrounding tissue was mostly in tact with cells in the outer annulus appearing viable. Biochemical analysis demonstrated greatly reduced glycosaminoglycan content in these compared to untreated discs. We have shown that bovine coccygeal discs, treated with proteolytic enzymes can provide a useful *in vitro *model system for developing and testing potential new treatments of disc degeneration, such as injectable implants or biological therapies.

## Background

There is currently a great deal of interest in developing new therapies to address degeneration of the intervertebral disc, due to the association of disc degeneration with low back pain. These therapies range from injectable synthetic nucleus replacements to biological approaches such as cell therapy [1,2]. Before being used in humans, any potential new therapies should be tested in model systems, either *in vitro *or *in vivo*. Animal models of disc degeneration are limited, with no ideal model [3]. Small animals such as rodents or rabbits have a different cell type and anatomy from that found in humans and large animals are expensive, do not develop the same pattern of degeneration as in humans, and some of these also have a different cell type.

Bovine caudal discs have been developed as a suitable model for many *in vitro *studies of the intervertebral disc, having similar physico-chemical properties to the human disc [4]. However, these bovine discs have such a high swelling-pressure that it is virtually impossible to inject any significant volume into them, thus they cannot be used for testing nuclear replacements unless they are modified. In this paper we describe a technique for developing an explant model of disc degeneration in the bovine caudal disc that would be suitable for testing injectable nucleus pulposus replacements. We have characterised the properties of the disc following enzymatic digestion which results in some similar changes as are seen in degeneration of the human intervertebral disc. This highlights its potential uses and limitations for testing novel therapeutic developments for degenerative intervertebral discs.

## Methods

### (i) Samples

Bovine tails were obtained from animals aged 18–30 months at a local abattoir within 1 hour of death. Before dissection tails were washed in 500 parts per million of chlorine to minimise risk of infection. Skin was removed and motion segments, 1.5 to 2 cm in diameter, were dissected from the tail, under sterile conditions. They were cleaned of soft tissues, including muscles and ligaments. The segments were washed thoroughly with sterile phosphate buffered saline (PBS, Invitrogen, Paisley) before washing for 10 minutes with standard culture medium (Dulbecco's modified Eagles medium (DMEM:F12, Invitrogen) supplemented with 500 μg/ml ascorbic acid (Sigma), 10% foetal bovine serum (PAA Laboratories, Yeovil), 0.05% Gentamicin (Invitrogen) and 0.005% Fungizone (Invitrogen)). Thirty eight motion segments (1.5–2.3 cm in diameter, mean 1.8 cm ± 0.3) were obtained from the central region of tails from approximately 12 animals.

### (ii) Enzyme Digestion

For induction of degeneration of the nucleus pulposus, motion segments were injected with i) 20 mg/ml of papain (18 U/mg protein, Sigma) in buffer (0.01 M cysteine hydrochloride, 0.5 mM EDTA, 0.2 M sodium acetate, pH 6), ii) trypsin, used at 1, 5, 10 or 20 mg/ml trypsin in PBS (12,400 U/mg protein Sigma), or iii) untreated, with nothing injected (as 'controls'). It was difficult to be accurate in recording the exact volume injected, due to subsequent back pressure but it was estimated that 70–100 μl was used per treatment. Motion segments were cultured in standard culture medium at 37°C in a 5%CO_2 _humidified atmosphere. At day 1 the action of the trypsin was stopped by injection with FBS; the medium was changed on all the motion segments at day 1 and then every 3 or 4 days. Three samples (randomised from different animals) for each treatment were harvested at 1, 2 and 3 weeks at which time they were X-rayed, cut in half sagittally and photographed. One half was processed for histology and the other was used for biochemical assessment. In addition 3 untreated discs were excised and analysed similarly to provide control baseline values ('normals').

The disc dimensions (circumference, diameter and height) were measured in a separate sample of 8 motion segments before (V_0_) and after (V_1_) digestion with papain for one week. These measurements were used to make an estimate of disc volume. Disc volume used to give an indication of the degree of swelling which might occur during the incubation.

### (iii) Radiography and histology

Intact motion segments were injected with a radio-opaque solution (Omnipaque, Amersham, Buckinghamshire) before X-raying (32 kV for 80 seconds exposure) and then dissected using a band saw. The volume injected was recorded. One half of the motion segment was fixed in formol saline for 24 hours before transferring to decalcifying solution (10% formic acid in formalin) for 1–2 weeks until decalcification was complete (as assessed with radiographs). Samples were then embedded in paraffin wax and 4 μ thick sections were cut and stained with either haematoxylin and eosin (H&E) or toluidine blue [5]. Morphology was assessed by standard light microscopy using both bright and polarised light.

### (iv) Biochemical assessment

Bone was removed from the cranial and caudal aspects of the remaining half motion segment. The central half of the disc was cut into 2 columns, which were then cut into approximately 1 mm thick slices from the outer annulus, through to the mid nucleus to provide a profile of glycosaminoglycan (GAG) and water contents. The pre-weighed slices of disc were dried to a constant weight at 60°C and the water content calculated as percentage of total wet weight.

The dried disc slices were then digested with 1 ml papain solution (2.5 mg of papain in 20 ml of 0.01 M cysteine hydrochloride, 0.5 mM EDTA, 0.2 M sodium acetate, pH 6) at 60°C for 4 hours. The GAG content was measured using a dimethylmethylene blue (DMB (Serva)) assay [6], modified to a 96 well plate method.

## Results

### (i) Morphology

**Figure 1:**
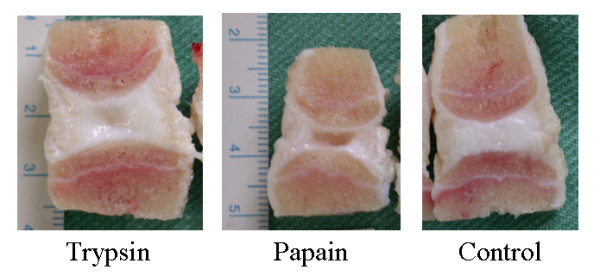
Macroscopic appearance of dissected motion segments after incubation for 1 week with 20 mg/ml of trypsin, papain or no enzyme (control).

**Figure 2:**
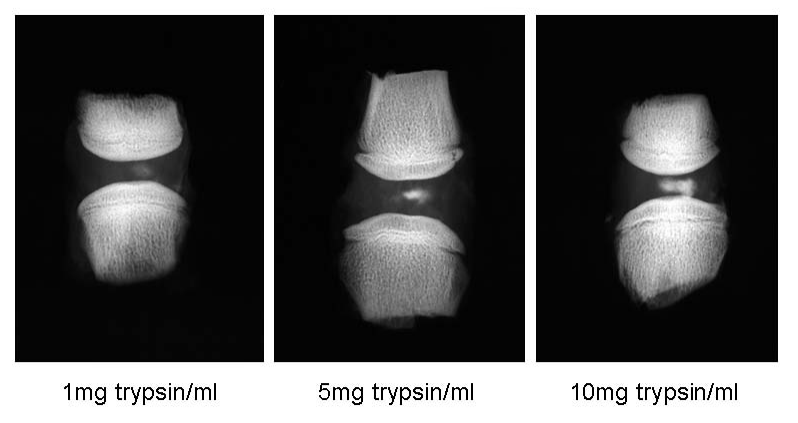
Radiographic images of motion segments injected with radio-opaque medium 72 hours after incubation with 1, 5 or 10 mg/ml trypsin.

Macroscopic changes to the structure of the enzyme treated discs were visible by the naked eye on dissection of the motion segments (Figure 1). The integrity of the control discs appeared intact at all time points; in contrast, all enzyme treated discs had cavities in the centre. This alteration was restricted to the nucleus pulposus for trypsin treated discs at all time points and at weeks 1 and 2 for papain-treated. At 3 weeks after papain injection, however, the disruption extended into the annulus for all samples so treated. Radiograph imaging of motion segments injected with radio-opaque dye demonstrate that the amount which could be injected into the centre of the disc increased with the amount of enzyme used (Figure 2), occupying approximately 10–25% of the disc volume after a week.

**Figure 3:**
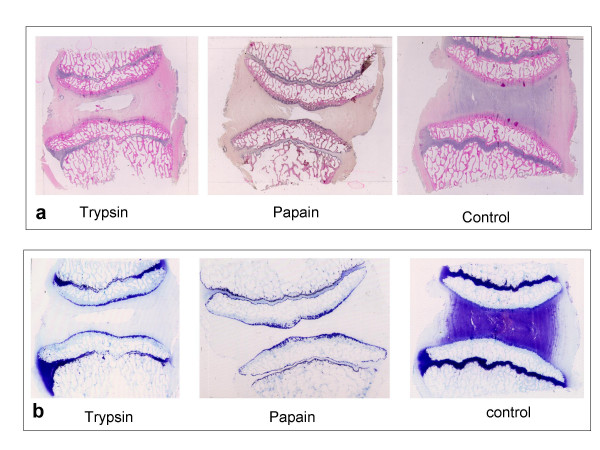
(a) H&E stained sections at 3 weeks post-incubation with 20 mg trypsin, 20 mg papain or no enzyme (control). (b) Toluidine blue stained sections demonstrating severe loss of metachromasia from all of the disc in enzyme treated discs compared to non-enzymatically digested, where loss is diminished and only in the outer annulus.

**Figure 4:**
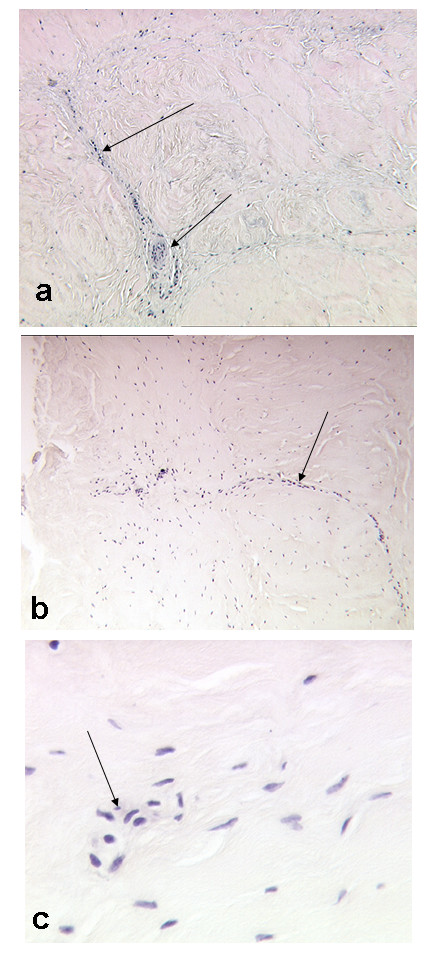
H&E stained sections of the annulus of discs showing blood vessels (arrows) and disc cells 2 weeks after incubation. (a) control discs (×40 mag.), (b) trypsin treated (×40 mag.) and (c) trypsin treated at higher magnification (×250).

These changes were also seen microscopically with central fissures seen clearly on all enzyme treated discs (Figure 3a). There were obviously no cells within the nuclear region which was digested, but in the surrounding matrix the cells were generally healthy in appearance (though no cell viability measurements were made) and occurring at a similar density as in the controls. Toluidine blue stained sections of untreated control discs demonstrated some loss of metachromasia, particularly in the outer annulus. However, sections of enzyme treated discs, both those treated with papain and trypsin, showed severe and much greater loss of metachromasia at all time points (Figure 3b). Control discs, incubated for 3 weeks had little metachromasia in the outer annulus; this pattern is similar to that of normal, freshly obtained discs, reflecting the lower GAG content found in this region. Blood vessels were seen in the outer annulus fibrosus of both enzyme treated and untreated discs. The morphology of these, the cells within them and the disc cells themselves appeared normal, even after 3 weeks of incubation (Figure 4).

The dimensions of the motion segments changed little during the enzymatic digestion and incubation. The original estimated volume of the disc (V_0_) was 1.10 ± 0.17 cm whereas 1 week later (V_1_) it was 1.11 ± 0.16 cm (n = 8). The volume which could be injected was 136 ± 44 μl (n = 8), ie 12% of the volume of the disc.

### (ii) Biochemistry

**Figure 5:**
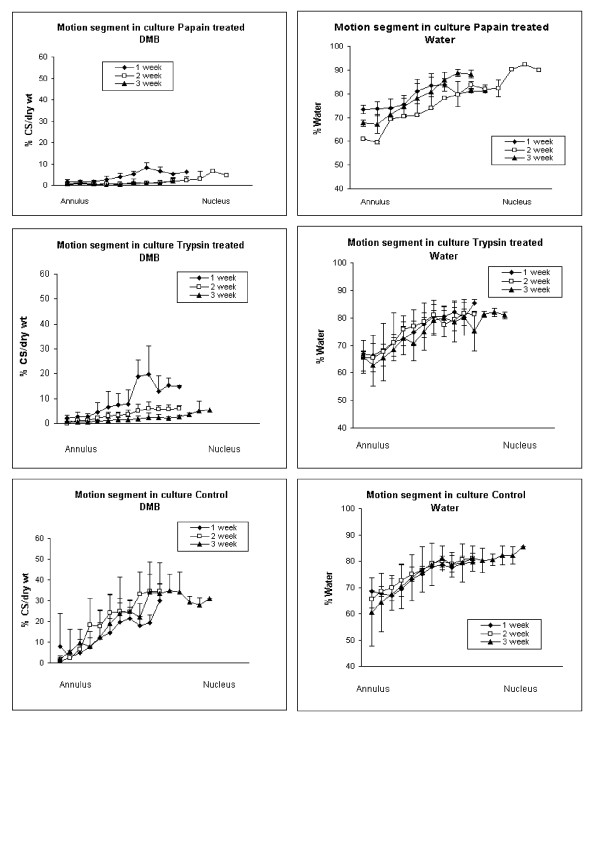
The profile from outer annulus to the central nucleus pulposus of (a) water and (b) GAG contents of fresh normal, bovine disc (i.e. not injected with enzyme or cultured).

The water and GAG contents of untreated, fresh (normal) bovine discs was similar to the control discs and, as reported many times previously, higher in the central nucleus pulposus than in the annulus fibrosus. Profiles of water content showed no significant difference from those in any of the discs which had been enzymatically digested (at any time points; Figure 5). The profiles of GAG contents, in contrast, were much lower for both enzymes used, but were particularly reduced for papain treated discs, which all had a GAG content lower than 10% CS/dry weight (compared to 15–30% of normal discs in the nucleus). Trypsin treated discs reached this level 2 weeks after the enzyme had been introduced.

## Discussion

Any new therapy developed for the treatment of intervertebral disc degeneration to alleviate or delay the onset of associated back pain must go through the inevitable series of testing prior to human trials. Models of disc degeneration have severe limitations [3] with none providing the ideal test environment. Certain properties, for example cytotoxicity, can be carried out on cell cultures. However, disc cells isolated from their extracellular matrix have a completely different morphology and size than when retained within it [7], such that explant models are more likely to reflect the *in vivo *situation than isolated cell models. Whilst the use of explant models will not preclude the complete use of whole animal *in vivo *models, they may be able to answer many of the questions which have to be asked and minimise the number and hence reduce the investment in cost and ethics otherwise required to take a new product to clinical trials and the marketplace.

There are already several *in vitro *models described in the literature which, although they are characterised by additional techniques (eg cell number and viability, immunohistochemistry, *qRT-PCR*) to those used in this study, many involve small species, for example mouse [8], rat [9,10] or rabbit [11]. These species have inherent limitations of size and notochordal cells within the nucleus pulposus region. More recently larger discs from bigger animals such as sheep and cattle have been cultured as explant models with the possibility of studying mechanisms of disc degeneration [12,13]. Likewise several models have utilised enzyme digestion of the extracellular matrix (summarised in reviews [4,14]. Many of these were developed to mimic and understand better the physiological response to the treatment of chemonucleolysis. More recently there is renewed interest in this as a clinical treatment, with chondroitinase ABC as an alternative enzyme. Imai et al [15] use this in a rabbit model of chemonucleolysis, whilst it has also been suggested to be useful for a model of degeneration in mice[16]. Antoniou et al [17] have used different enzymes to examine the effect of degradation of individual matrix components (for example, collagen or proteoglycans) on magnetic resonance imaging parameters. Little has been detailed, however, on the GAG profile, which is probably the most important biochemical parameter relating to disc degeneration.

There are real physical limitations of injecting any significant volume into a healthy disc (though small volumes such as might be used for testing growth factor or even cell therapy can obviously be introduced). In contrast, in the clinical setting where it is envisaged that newly designed implants might be injected into a degenerate human lumbar intervertebral disc, it can often be easy to inject volumes of at least 2 ml of a substance (i.e. approximately 10% of its volume). Hence there is a requirement for a model system of a degenerate disc which is larger than the animal discs most commonly used in such tests and which has properties more similar to those of the human disc. The bovine coccygeal disc provides such samples, with similar properties in terms of composition, swelling pressure and metabolism to those seen in human discs [4]. We have shown in this study that breakdown and loss of GAGs can be induced, sufficient to inject a potential nuclear replacement for subsequent testing. (Several nuclear replacements are being investigated by other groups, for example, NuCore^® ^Injectable Nucleus (Spine Wave Inc, Shelton, CT, USA) and the BioDisc™ (Cryolife Inc, Kennesaw, GA, USA)).

There have been some studies examining explant culture with and without the vertebral endplates attached, indicating that bovine discs without endplates and ovine with endplates could maintain viability for up to 1 week [12,13]. The vertebral bone (and ligamentous tissue) certainly seemed to be an advantage in the present study in that swelling was severely restricted, with no significant or obvious swelling of the whole motion segment, although it is likely that the similar water content seen in the treated and control discs was due to lack of restraint against swelling via musculature, ligaments etc such as would occur in vivo. In addition, the enzymes used to treat the motion segments did not appear to have affected the histology of the remaining surrounding tissue greatly or any differently from that seen in the untreated controls. Both trypsin and papain created a 'space' allowing material to be injected, but trypsin may be preferable on a cost-basis and is certainly much cheaper than other enzymes such as chymopapain or collagenase. Likewise, there was little difference between time-points studied, particularly for papain, hence this would be recommended to use for the shortest interval.

This model could have several uses for testing newly developed products. These could include application systems, mechanical properties of implants or their adhesive and interaction properties. Indeed, preliminary data has been obtained with this model, whereby an injectable gel has been inserted and tested under either hydrostatic load (approximately 4 kg for 2 hrs) or cyclic loading (4 kg, 600 cycles at a frequency of 1 Hz; Dr S Sarit, personal communication). In addition to testing implants, they could also be used to assess biological approaches and, for example, monitor injected cells. When developing new therapies, the choice of the ideal test system depends very much on the questions being asked. In this study we have shown that the use of a general proteolytic enzyme together with a bovine coccygeal explant system can provide a means of producing a cheap, reproducible system suitable for testing some of the new generation of injectable implants which are being developed in the spine field.

## Conclusion

Several injectable novel therapies are being developed worldwide for treating degeneration of the intervertebral disc associated with back pain. As with all such developments these require testing and evaluation in the laboratory prior to taking to the clinic. Unfortunately 'normal' discs have a high swelling pressure and resist injecting any volume of substance into. Hence we have developed an explant model which mimics some aspects of disc degeneration and would be suitable for testing the new generation of injectable therapies for treating degenerate discs in humans.

## Competing interests

The author(s) declare that they have no competing interests.

## Authors' contributions

SR: preparation of manuscript, experimental design, interpretation and obtaining funding; JM: experimental design and laboratory work; SS: discussion on application of model; JU: experimental design and guidance and obtaining funding. All authors read and approved the final manuscript.

## Pre-publication history

The pre-publication history for this paper can be accessed here:


